# Phenolic acid-degrading *Paraburkholderia* prime decomposition in forest soil

**DOI:** 10.1038/s43705-021-00009-z

**Published:** 2021-03-25

**Authors:** Roland C. Wilhelm, Christopher M. DeRito, James P. Shapleigh, Eugene L. Madsen, Daniel H. Buckley

**Affiliations:** 1 School of Integrative Plant Science, Bradfield Hall, Cornell University, Ithaca, NY USA; 2 Department of Microbiology, Wing Hall, Cornell University, Ithaca, NY USA

**Keywords:** Microbial ecology, Forest ecology, Soil microbiology, Biogeochemistry

## Abstract

Plant-derived phenolic acids are catabolized by soil microorganisms whose activity may enhance the decomposition of soil organic carbon (SOC). We characterized whether phenolic acid-degrading bacteria enhance SOC mineralization in forest soils when primed with ^13^C-labeled *p*-hydroxybenzoic acid (*p*HB). We further tested whether *p*HB-induced priming could explain differences in SOC content among mono-specific tree plantations in a 70-year-old common garden experiment. *p*HB addition primed significant losses of SOC (3–13 µmols C g^−1^ dry wt soil over 7 days) compared to glucose, which reduced mineralization (-3 to -8 µmols C g^−1^ dry wt soil over 7 days). The principal degraders of *p*HB were *Paraburkholderia* and *Caballeronia* in all plantations regardless of tree species or soil type, with one predominant phylotype (RP11^ASV^) enriched 23-fold following peak *p*HB respiration. We isolated and confirmed the phenolic degrading activity of a strain matching this phylotype (RP11^T^), which encoded numerous oxidative enzymes, including secretion signal-bearing laccase, Dyp-type peroxidase and aryl-alcohol oxidase. Increased relative abundance of RP11^ASV^ corresponded with higher *p*HB respiration and expression of *p*HB monooxygenase (*pobA*), which was inversely proportional to SOC content among plantations. *pobA* expression proved a responsive measure of priming activity. We found that stimulating phenolic-acid degrading bacteria can prime decomposition and that this activity, corresponding with differences in tree species, is a potential mechanism in SOC cycling in forests. Overall, this study highlights the ecology and function of *Paraburkholderia* whose associations with plant roots and capacity to degrade phenolics suggest a role for specialized bacteria in the priming effect.

## Introduction

Forest soils are rich in plant-derived phenolic acids which represent a sizeable proportion of fast-cycling, low-molecular weight soil organic carbon (SOC),^1^ estimated at between 10 and 4000 mg · kg^−1^ dry organic layer soil.^2–5^ Concentrations of soil phenolics fluctuate as a function of plant inputs and phenolic acid-degrading activity.^4,6–10^ The cycling of soil phenolics has the potential to accelerate decomposition according to research linking phenolic acids, and phenol oxidase activity, to the soil priming effect.^11–16^ The priming effect refers to the enhanced decomposition of SOC which can occur when an influx of new C stimulates microbial activity, which is a fundamental process in terrestrial C-cycling.^17,18^ The activity of phenolic acid-degrading populations appear to be especially impactful, priming a greater loss of SOC (per unit biomass) than glucose or cellobiose.^14^ In this light, the low phenolic content of older accumulations of SOC^19,20^ may reflect a subsidence in the priming activity fueled by phenolics. Accordingly, we would expect to find a link between phenolic acid-degrading populations, soil priming, and SOC accrual in nature. However, this link has not yet been tested and the identity and ecology of these populations, and the mechanisms behind their priming activity, remain poorly described.

The primary sources of phenolic acids to soil are litter leachate, root exudates, and the decomposition products of lignin-rich plant residues. The composition of soil phenolics depends on the plant community, environmental conditions, and soil properties,^21–24^ with broad differences between coniferous and deciduous forests.^2,25–27^ *p*-hydroxybenzoic acid (*p*HB) is one of the most abundant phenolic acids found in plant litter, tree root exudates, and soil, ranging in concentrations from 0.1 to 50 mg · kg^−1^ dry wt in bulk soil^4,5,7,28–30^ which can be three to four-fold higher in the rhizosphere.^31^ Plant *p*HB content was found to correlate with the strength of priming^13^ and benzoic acid, an abundant root exudate with similar properties as *p*HB, can induce priming.^15^ To compare differences in *p*HB sources, and corresponding activity of *p*HB-degrading populations, we conducted an experiment using a 70-year-old common garden with plantations of a coniferous tree species (red pine) and two deciduous tree species: one leguminous (black locust) and one non-leguminous (sugar maple).^32^

Most phenolic acid-degrading microorganisms isolated from soil are fast-growing members of the *Beta-* and *Gammaproteobacteria* (Table S1).^33–36^ Rapid growth may be essential for metabolizing phenolics in soil, which quickly adsorb to surfaces of minerals and organic matter, reducing their bioavailability and promoting SOC accumulation.^31,37–39^ Though a culture-independent survey of phenolic acid-degrading microbes has not yet been made in forest soils, metagenomic analyses of lignin-degrading populations implicate *Alphaproteobacteria* (*Rhizobiales*, *Sphingomonadales* and *Caulobacterales*), *Betaproteobacteria* (*Burkholderiaceae*) and *Actinobacteria* (*Nocardiaceae*, *Frankiaceae*, *Streptomycetaceae* and *Microbacteriaceae*).^40–42^ Populations of *Alpha*- and *Betaproteobacteria* are abundant during white-rot decay of wood, where phenolic acid concentrations are high;^43,44^ and in the rhizosphere, where roots exude phenolic acids to facilitate plant-microbe interactions.^45,46^ Accordingly, we expect the phenolic-acid degrading populations that mediate priming will belong to these three bacterial classes, representing a narrower diversity of taxa than observed during glucose-induced priming.^47^

The priming effect can be generated by a variety of mechanisms that interact in a context-dependent manner.^48,49^ In certain cases, priming coincides with an increased oxidative potential of soils.^11,12,50^ Measures of potential oxidative activity may therefore serve as useful proxies for priming responses. *p*HB oxidation is catalyzed by *p*-hydroxybenzoate 3-monooxygenase (*pobA;* EC 1.14.13.2) as part of the peripheral pathways for the degradation of aromatics.^51^ PobA catalyzes an essential step in the degradation of phenolics, which can be the rate limiting step in the degradation of certain polyphenolics in soil.^52^ Furthermore, the expression of *pobA* is responsive to environmental conditions, evidenced by its upregulation in *Burkholderia multivorans* grown in soil versus laboratory media.^53^ Consequently, the expression of *pobA* may provide a proxy for the activity of phenolic acid-degrading soil populations and any potential priming effects.

In previous research, we have shown that an abundant aromatic acid in plant root exudates (benzoic acid) can prime decomposition mediated by the activity of phenolic-degrading bacteria.^15,54^ Our present study sought to test the link between phenolic acid-degrading populations, soil priming, and SOC accrual in a 70-year-old common garden experiment^32^ and the mechanism by which their metabolism controls soil priming. We tested whether *p*HB primes microbial activity that enhances SOC decomposition, and whether this phenomenon corresponded to differences in SOC content among monospecific tree plantations. We measured *p*HB degradation in the field and its capacity to prime SOC mineralization in a microcosm experiment. *p*HB-degrading populations were characterized using 16S rRNA amplicon sequencing, stable isotope probing (SIP) with ^13^C-*p*HB, the expression of *pobA*, as well as physiological and genomic characterization of *p*HB-degrading isolates. We hypothesized that the activity of *p*HB-degrading populations will prime the loss of SOC and that differences in this activity will correspond to variation in SOC content in forest soils. Specifically, we predicted (i) a positive relationship between the activity of *p*HB-degrading bacteria and SOC priming and (ii) an inverse relationship between SOC content in forest soils and *p*HB-degrading activity. Our microbe-centric approach supposes that the ecological and functional traits of phenolic acid-degrading bacteria are mechanistically important to soil priming and in environmental processes that govern the fate of SOC.

## Material and methods

### Description of field site

We studied *p*HB-degradation and the priming effect in soils from a 70-year old common garden experiment consisting of uniform 0.4 ha plots planted with monocultures of 13 tree species spanning types typical of northeastern USA (Turkey Hill Forest Plantation, Dryden, NY).^32,55^ The site was reforested following a period of over 100 years of agricultural use. We sampled plantations of sugar maple (“SM”, *Acer saccharum* Marsh; 42.451137, -76.420519), red pine (“RP”, *Pinus resinosa* Ait.; 42.450945, -76.420638), and black locust (“BL”, *Robinia pseudoacacia* L.; 42.451818, -76.420614) to capture variation in sources of soil phenolics. Plantations were chosen to include representatives of coniferous and deciduous tree species and legume-forming (black locust) and non-leguminous species (sugar maple). Trees were grown in typic Fragiochrept soils (inceptisols) belonging to either the Mardin B (“MaB”; Typic Fragiudepts) or Lordstown channery silt loam C soil series (“LnC”; Typic Dystrudepts). Our study examined a total of five combinations of tree species + soil type, which we have termed ‘ecoplots’, that encompassed a gradient in total soil organic matter: BL_MaB_ (21.9% w/w) > SM_MaB_ (14.5%) > BL_LnC_ (14.0%) > RP_LnC_ (12.75%) > SM_LnC_ (10.2%). There were no plantations of RP in MaB soil. Earthworms populations, which can accelerate decomposition,^56,57^ did not correspond with trends in SOC in our plantations.^58^ Details on the site and three separate field sampling campaigns are provided in the Supplementary Methods. The concentration of bioavailable elements in soils was determined using the modified Morgan extraction procedure^59^ by the Cornell Nutrient Analysis Laboratory. Estimates of soil organic matter were made according to the loss-on-ignition method.

### pHB-induced soil priming

Microcosms were prepared with soils from BL_LnC_, RP_LnC_ and SM_LnC_ to test whether *p*HB degrading activity induced priming and to identity and link active populations with those observed in the field experiment. Microcosms were prepared and amended with the following treatments: (i) water-only control, (ii) ^13^C-glucose, (iii) ^13^C-*p*HB, (iv) ^13^C-*p*HB + PobA inhibitor (4-hydroxy-3-iodobenzoate) and (v) a PobA inhibitor-only control (overview in Fig. S1). The inhibition of PobA was performed to measure the direct contribution of PobA activity to priming. An additional set of microcosms was used to test whether priming could be induced by artificially increasing the abundance of a phenolic acid-degrading isolate (RP11^T^), corresponding to main *p*HB-degrading phylotype in field data (RP11^ASV^; details in Supplementary Methods). Microcosms were prepared using fresh soil collected from the upper 5 cm of a 20 × 20 cm sampling plot, which was sieved to remove root and litter (2-mm), homogenized, and pre-incubated for 10 days. Sieving, homogenization, and pre-incubation were necessary to reduce soil SOC heterogeneity and limit variability in the concentration of labile SOC at the time of sampling. After pre-incubation, soils were air dried for 48 h in a sterile biosafety hood. Ten grams of dry soil were added to 120-mL serum vials and wetted during treatment application to 60% water holding capacity. Glucose and *p*HB were added at a concentration of 0.5 mg C per g dry wt soil. Each treatment was run in quadruplicate per ecoplot. Isotopically labeled substrates (17.5 atom % ^13^C) or RP11^T^ cells (99 atom % ^13^C) were used to distinguish between the CO_2_ derived from amendment versus SOC. The isotopic composition of amendments was confirmed by EA-IRMS by the Cornell Stable Isotope Laboratory. Headspace sampling was performed every 24 hrs for a week with the headspace exchanged with filtered air after each sampling. The quantity of ^12^CO_2_ (m/z 44) and ^13^CO_2_ (m/z 45) was analyzed using GC/MS (Shimadzu GCMS-QP2010S) with a set of standards ranging from 1000 to 40,000 ppm CO_2_. After 7 days, sub-samples of 0.5 g of wet wt soil were stored at −80 °C for RNA-based quantification of *pobA* gene abundance and bacterial community composition. Headspace CO_2_ measurements continued at reduced frequency for a total of 26 days. All methodological details are available in the Supplementary Methods.

### pHB degradation in field soil

To test whether phenolic acid-degrading activity differed among ecoplots, *p*HB mineralization rates were measured in field soils in an experiment which also served to identify the active in situ populations via DNA-SIP. Sampling took place in three randomly selected plots (20 × 20 cm) per ecoplot. The litter layer was cleared to expose the O_a_ / A-horizon soil and stainless-steel chambers (radius 1.25 cm) were driven into the soil at an even depth (2 cm). Photographs of field sampling are provided (Fig. S2). A thin layer of surface soil was evenly wetted by dripping 150 µl of either ^13^C_7_-labeled *p*-hydroxybenzoic acid (99% atom ^13^C; Sigma-Aldrich) or unlabeled control (‘^12^C’; Sigma-Aldrich) at a concentration of 1000 ppm (6.9 and 7.3 mM, respectively). Chambers were immediately enclosed with rubber septa and ^13^CO_2_ concentrations were measured in the headspace at five sampling timepoints over a period of 27 h. At each time point, 2.5 mL of headspace was sampled and stored in evacuated 2-mL vials prior to analysis with a Hewlett Packard 5890 gas chromatograph (Wilmington, DE) equipped with a 5971A mass selective detector (details in Supplementary Methods). Net ^13^CO_2_ was determined by subtracting average natural abundance ^13^CO_2_ in ^12^C controls from the total ^13^CO_2_ respired in ^13^C-*p*HB-amended soils. Following the incubation period, ~0.5 g of soil was collected from the upper 1 cm of the dosed soil, transported on ice and stored at −80 °C for use in DNA-SIP.

### Identifying pHB-degrading populations

Nucleic acids were extracted from 0.5 g of soil using the modified phenol-chloroform method from^60^ following bead-beating at 5.5 m·s^−1^ for 2.5 min (Bio Spec Products, Santa Clara, CA, USA) in 2-mL Lysing Matrix E Tubes (MP Biomedicals, OH). For the DNA-SIP experiment, DNA was extracted from ^13^C- and ^12^C-*p*HB amended field soils and ^13^C-enriched DNA was separated from unlabeled DNA by CsCl density gradient ultracentrifugation as previously described.^61,62^ Twenty 200-µL fractions were collected from each CsCl gradient and 16S rRNA gene amplicon sequencing libraries were prepared for fractions F_1-2_ (pooled), F_3_, F_4_, F_5_, F_6_, F_7_, F_8_, F_9-10_, and F_11-12_ (spanning CsCl buoyant density of 1.77–1.70 g·mL^-1^). RNA was coextracted with DNA for quantifying the expression of *pobA*, in field soils and microcosms, and for profiling the active populations in microcosms targeting 16S rRNA. Complementary DNA (cDNA) was synthesized from DNAse-treated RNA extracts using SunScript reverse transcriptase (Expedeon, San Diego USA). Bacterial community composition was assessed by amplification of the V4 region using polymerase chain reaction (PCR) with dual-indexed barcoded 515f/806r primers.^63^ PCR was performed in duplicate and pooled, and PCR products were purified and normalized to a standard concentration prior to sequencing on three lanes of Illumina MiSeq (2 × 250 paired end) at the Cornell Biotechnology Resource Center. Amplicon libraries were deposited in the European Sequence Archive under the BioProject accession PRJEB23740. Following sequencing processing (described below), amplicon sequence variants (“phylotypes”) were designated as *p*HB-degraders if their relative abundance was at least eightfold greater in heavy CsCl fractions (F_3_-F_8_) in ^13^C-enriched versus ^12^C-control amplicon libraries, after variance stabilization by DESeq2 (v. 1.24.0).^64^ Phylotypes with increased relative abundance in rRNA pools in the microcosm-based priming experiment were identified by indicator species analyses using the R package “indicspecies”.^65^ Full details on methods and controls for DNA extractions, cDNA synthesis, PCR conditions and isopycnic gradient centrifugation are available in the Supplementary Methods.

### Isolation, genome sequencing, and functional description of pHB degraders

*p*HB degrading isolates were obtained from red pine and sugar maple LnC soil by serial dilution. Diluted cells were plated onto mineral salts media containing 3 mM *p*HB (MSM-*p*HB) as the sole carbon source and incubated at room temperature. Colonies appeared after 3 days and were isolated by streaking on MSM-*p*HB and stored in 20% glycerol (v/v) at −80 °C. Genomic DNA was extracted from isolates and isolates were identified by Sanger sequencing of the 16S rRNA gene (27f/1492r).^66^ The *p*HB degrading activity of isolates was validated based on protocatechuate production with GC/MS (details in Supplementary Methods). An isolate, RP11, matched one of the predominant *p*HB-degrading phylotypes in amplicon libraries and its genome was sequenced using a quarter lane of Illumina MiSeq (2 × 250bp). The sequencing reads and genome assembly are available from the NCBI (BioProject PRJNA558488). The strain was subsequently described as *Paraburkholderia madseniana* RP11^T^ (DSM 110123) and exhibited growth on several phenolic acids including *p*-coumaric and phthalic acids.^54^ Details on the assembly, calculation of average nucleotide identity (ANI) and functional gene annotation are available in the Supplementary Methods.

### Quantifying pobA expression

Custom PCR primers were designed to target a 75-bp fragment of the “*pobA1*” gene identified in the genome of isolate RP11^T^: *pobA1*_f (5’-AGA TCG AAT CCA CCA TCC GC-3’) and *pobA1*_r (5’-TTC AAA GCC GTG ATG CAA CG -3’) using NCBI’s Primer-BLAST software.^67^ Details of primer design and validation and RT-qPCR conditions are provided in the Supplementary Methods. In brief, the quantification of *pobA* and 16S rRNA transcripts (primers 341f/534r) was performed by RT-qPCR using an AB7300 Real-Time PCR system (Applied Biosystems).^68^ *pobA* gene expression was assayed using triplicate soil cores per ecoplot in the field. Cores were taken with 3-mL plastic syringe barrels and immediately extruded into 10-mL serum bottles and dosed with 75 µL of ^13^C-labeled *p*HB (1000 ppm). Expression levels were determined in RNA extracted from 1 g of soil following a 24-h incubation in field and following a 7-day period in microcosms.

### Prevalence of pobA in environmental metagenomes and bacterial genomes

To gain insight into the diversity of bacteria capable of *p*HB-degradation and to identify environments with the greatest potential activity, we conducted surveys of the prevalence of *pobA* in publicly available metagenomic (*n* = 14,139) and genomic datasets (*n* = 11,643) through the IMG/ER portal.^69^ The number of *pobA* copies per genome and the relative abundance in metagenomes were determined using the KEGG function search tool (March 19th, 2019). Taxonomic classifications were based on those provided by IMG/ER and the environmental source of metagenomes was attributed to the ‘habitat’ descriptor. Custom *pobA* Hidden Markov Models (HMMs) were generated from an alignment of homologs present in all *Burkholderiaceae* genomes in the NCBI refseq_genomic database (*n* = 3991; as of June 20th, 2019). HMMs were developed for each of the seven *pobA* clades. A detailed description of all *pobA* analyses are provided in the Supplementary Methods and Supplementary Results, including information on enzyme structure and function and the phylogenetic trait depth of *pobA* paralogs (Table S2 and S3).^70^

### Data analysis and statistics

Amplicon libraries were quality processed using QIIME2 (v. 2017.9)^71^ with dependency on DADA2 (v. 1.2.0)^72^ to assign sequences to amplicon sequence variants (ASVs), referred to throughout as phylotypes. Taxonomic classification was performed using the QIIME2 “q2-feature-classifier” trained on the Silva database (nr_v132).^73^ Libraries were filtered to remove phylotypes present in reagent blanks or in low relative abundance (< 0.05% of all sequences) or few samples (minimum three samples). Estimates of species richness and Pielou’s evenness were calculated using rarified data (*n*_min_ = 9140 per sample). All other analyses were performed on counts normalized to library totals (counts per thousand). All statistical analyses were performed in R (v. 3.4.0; R Core Team, 2018) with general dependency on the following packages: ggplot2 (v. 3.2.1)^74^ and phyloseq (v. 1.22).^75^ Variable selection was performed to identify the soil physicochemical parameters contributing most to betadiversity was performed using stepwise regression with the “step” function in R. Non-metric multidimensional scaling (NMDS) and perMANOVA were performed with parameters that contributed the most to variation using the R package “vegan” (v. 2.5-6).^76^ Betadiversity was calculated for whole amplicon libraries using Unifrac distances based on a rooted tree produced using the QIIME2 wrapper “q2-phylogeny” for MAFFT (v. 7.407)^77^ and FastTree (v. 2.1.10).^78^ Selected parameters were then fit to the NMDS with the ‘envfit’ function in vegan. The occurrence of *Paraburkholderia* in studies of phenolic acid-induced priming, root associations and lignin decomposition was determined by re-analyzing published 16S rRNA gene data using the aforementioned workflow (see Supplementary Results). All analyses are reproducible using R scripts in the Supplementary Data.

## Results

We measured the priming effect in soil microcosms using ^13^C-labeled substrates (*p*HB and glucose) to differentiate the respiration of substrate from native SOC, while monitoring changes in microbial community composition and *pobA* expression. The activity and composition of *p*HB-degrading bacteria were determined in field soils by monitoring *p*HB respiration, *pobA* expression and performing DNA-SIP. This combined approach enabled us to link *p*HB-degrading populations with priming activity and track their activity in the 70-year-old common garden plantations (“ecoplots”) which differed by tree species, soil type, and SOC content. Differences in the sources and composition of phenolics, according to tree species, were expected to promote variation in phenolic acid-degrading/priming activity and community structure, which was expected to reflect differences in total SOC content.

### pHB-induced soil priming in microcosms

**Fig. 1:**
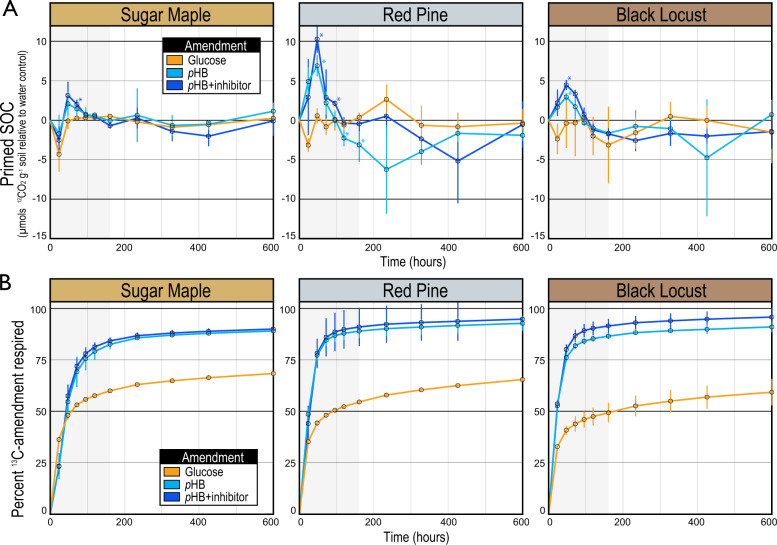
In soil microcosms, the enhanced mineralization of SOC is primed by the addition of *p*HB but not glucose. The addition of *p*HB caused positive priming (**A**), which corresponded with maximal respiration of ^13^C-*p*HB (**B**) during the first 7 days of incubation (shaded in gray). Primed SOC is a measure of the native SOC respired relative to water-only controls. Error bars correspond to standard deviations among replicates (*n* = 4). An asterisks denote time points where respiration of native SOC significantly differed from water-only control. Priming was assessed in LnC soils which varied in soil organic matter content (BL_LnC_: 14.0% > RP_LnC_: 12.7% C > SM_LnC_: 10.2%).

The addition of *p*HB primed the decomposition of significant amounts of SOC during the first four days of incubation, when rates of *p*HB respiration were highest (Fig. 1A). The majority of *p*HB had been mineralized by day 7 (Fig. 1), corresponding with the cumulative priming of 3.3 (SM_LnC_), 5.5 (BL_Lnc_), and 13 (RP_LnC_) µmols C g^−1^ dry wt soil, representing 3.6%, 4.5%, and 8.7% more SOC mineralization than soils that received water only. In contrast, glucose reduced SOC mineralization between −3 to −8 µmols C g^−1^ dry wt soil in the same period. *p*HB degradation and priming were significantly higher in RP_LnC_ than BL_LnC_ and SM_LnC_ (TukeyHSD; *p* < 0.05), while differences between BL_LnC_ and SM_LnC_ were not significant. *p*HB was respired more rapidly and to a greater extent (93–100% of amendment) than glucose (62–71%) in all ecoplots (Fig. 1B). Inhibition of *pobA*, while effective in pure culture (see Supplementary Results), was ineffective in soil, having no effect on *p*HB respiration or *p*HB-induced priming in soil microcosms (Fig. 1).

**Fig. 2:**
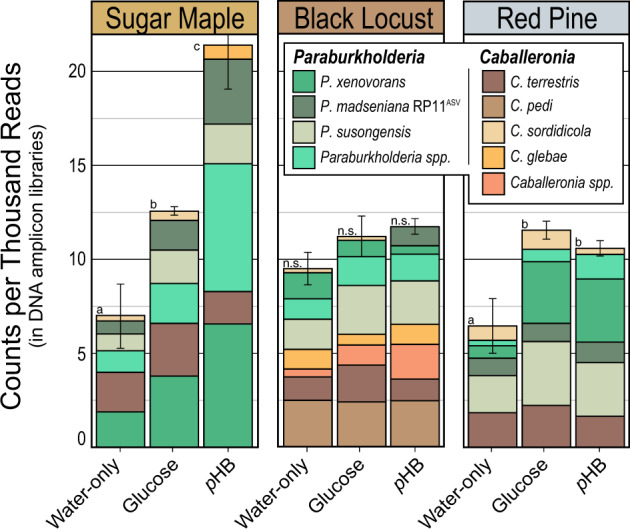
In soil microcosms, the relative abundance of *Paraburkholderia* and *Caballeronia* phylotypes increased when either *p*HB or glucose was added. Phylotypes were designated to a species group based on the top representative BLAST hit to type strains (100% identity). Several lesser abundant phylotypes were grouped as “*Paraburkholderia* spp.” and “*Caballeronia* spp”.

In soil microcosms, 70 bacterial phylotypes increased in relative abundance in response to glucose (*n* = 8), *p*HB (*n* = 10), water & glucose (*n* = 36) or glucose & *p*HB (*n* = 16; Table S4). Several phylotypes classified to *Paraburkholderia* and *Rhodanobacter* were consistently enriched in all microcosms supplied with *p*HB and glucose, respectively (Fig. 2; Table S4). Four of the seven phylotypes with the greatest response to *p*HB (relative abundance in *p*HB versus water-only) were *Paraburkholderia*, while the others were *Alicyclobacillus* (SM_LnC_), *Streptacidiphilus* (RP_LnC_) and an unclassified member of the phylum WPS-2 (RP_LnC_). The relative abundance of *Paraburkholderia* increased in response to *p*HB and glucose in all soils (Fig. 2), though this effect was not significant in BL_LnC_. In addition, *Paraburkholderia* responded more strongly to *p*HB than to glucose in SM_LnC_ soil (TukeyHDS; *p* < 0.01; Fig. 2). Transcripts of *pobA* were undetectable at day 7 (detection limit ~300 copies per µL; data not shown) consistent with *p*HB mineralization dynamics (Fig. 1A).

Soils amended with glucose, or with RP11^T^ cells, mineralized less SOC than those that received only water (i.e., “negative priming”), though the effect was statistically significant only in the latter treatment (Fig. S3). Amendment with RP11^T^ cells produced 5–14% less SOC mineralization when compared to controls. In all cases, negative priming was associated with steep increases in the relative abundance of *Streptomyces* and *Streptacidiphilus* phylotypes (Fig. S3; details in Supplementary Results).

### pHB-degrading activity of field soils

**Fig. 3:**
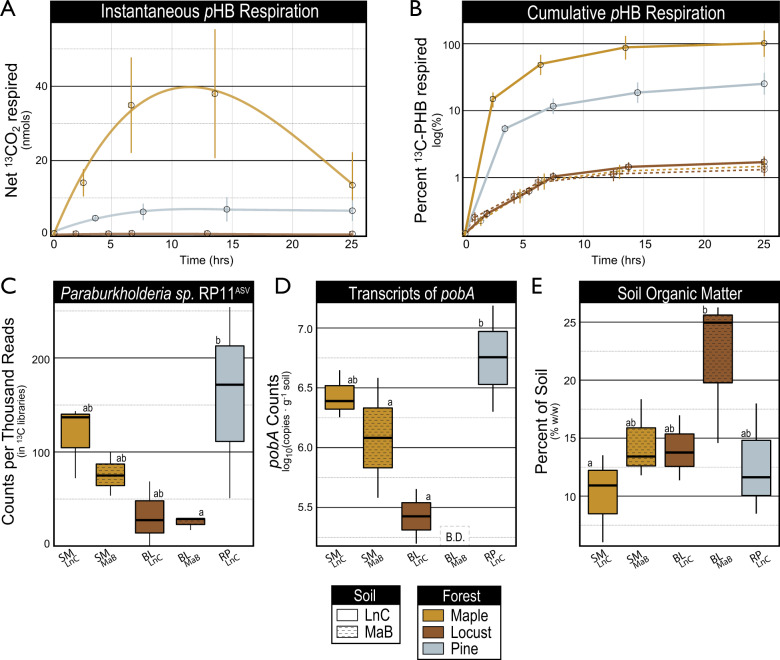
In situ measurements show that maximal rates of *p*HB respiration (**A**, **B**) are associated with high relative abundance of the RP11 phylotype (**C**), high levels of *pobA* transcripts (**D**), and low levels of soil organic matter (**E**). Error bars correspond to standard deviation (*n* = 3) and letters denote significant differences (*p* < 0.05) based on Tukey’s Honest Significant Difference tests (*n* = 3).

The respiration of *p*HB and expression of *pobA* were compared among ecoplots to test whether tree species (phenolic source) and soil type affected *p*HB-degrading activity. The amounts of *p*HB respired in situ differed considerably among ecoplots (Fig. 3). *p*HB was respired rapidly in SM_LnC_ and RP_LnC_ soils with maximum instantaneous rates observed after about 10 h (Fig. 3A, B). In contrast, *p*HB-degrading activity was relatively modest in SM_MaB_, BL_MaB_ and BL_LnC_, with substrate respiration plateauing at ~1.5–2% of the total C added to soil (Fig. 3B). SOC content was lowest in SM_LnC_ and RP_LnC_ where the highest rates of *p*HB respiration, *pobA* transcription, and the highest relative abundances of RP11^ASV^ occurred (Fig. 3C, D, E). These trends were not driven by total microbial biomass since RNA yield and 16S rRNA abundance were directly proportional to soil organic matter content (Fig. S4A, B).

### pHB-degrading populations

**Fig. 4:**
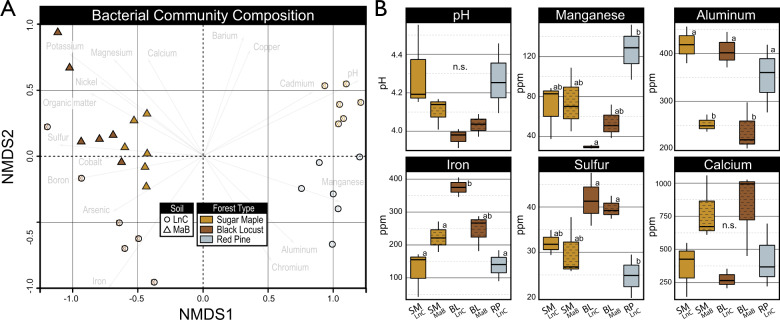
Variation in microbial community composition was driven by tree species composition and soil properties. NMDS ordination based on Bray-Curtis dissimilarity of 16S rRNA sequences from *p*HB-amended field soils indicate that soil series, tree species, and soil properties influence community composition (**A**). Soil physicochemical properties differed among ecoplots (B). In (**A**), community composition is represented by the average of all gradient fractions for ^13^C-labeled and ^12^C-control libraries for each ecoplot (*n* = 6). In (**B**), letters denote significant differences in soil properties (*p* < 0.05) based on Tukey’s Honest Significant Difference tests (*n* = 3). The hatching of box plots denotes the Mardin soil type. Measured concentrations correspond to the bioavailable elemental content of soils according to the modified Morgan extraction procedure. Comprehensive soil physicochemical properties are available in Table S6.

The active *p*HB-degrading populations were identified by 16S rRNA gene sequencing in field soil by DNA-SIP and in microcosm with RNA sequencing. Overall bacterial community composition in the field varied most by tree species (R^2^_perMANOVA_ = 0.25; *p* < 0.001) followed by soil type (*R*^2^ = 0.14; *p* < 0.001), illustrated by the NMDS ordination (Fig. 4A). Of all the soil properties, pH and aluminum content explained the most variation in community structure (*R*^2^_perMANOVA_ = 0.096 and 0.07, respectively; Table S5). BL forest soils had significantly lower pH, higher iron and sulfur, and lower cadmium and manganese than other forest types, while LnC and MaB soil types differed by aluminum and calcium content (Fig. 4B; Table S6). These data show that major differences in soil properties and microbial communities had developed among ecoplots during the 70-year period since planting.

**Fig. 5:**
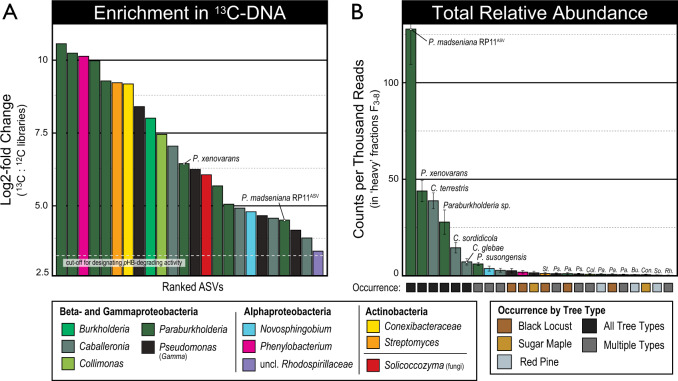
*Paraburkholderia* phylotypes were highly abundant in ^13^C-DNA recovered from DNA-SIP experiments in which ^13^C-*p*HB was added to field soils. *Paraburkholderia* represented one-third of all phylotypes enriched significantly in ^13^C-DNA as determined by their log_2_-fold differential abundance relative to corresponding ^12^C-DNA controls shown in (**A**). *Paraburkholderia* and *Caballeronia* phylotypes identified in (**A**) comprised the seven most relatively abundant phylotypes in ^13^C-DNA, and these phylotypes were found in all tree plantations, as shown in (**B**). The two most abundant ^13^C-labeled phylotypes matched *P. madseniana* RP11^T^ and *P. xenovorans*, respectively (**B**). Bars are colored by taxonomic classification and labeled with an abbreviated taxon name where bar coloring is obscured. Error bars correspond to standard deviation (*n* = 3). Phylotypes were designated to a species group based on the top representative BLAST hit to a type strain (100% identity). Details on the rank and relative abundance of phylotypes are available in Table S7.

Despite these differences, we observed that the same six *Burkholderiaceae* phylotypes dominated in situ *p*HB degradation in all ecoplots (Fig. 5A). Bacterial communities profiled from the ^13^C-labeled DNA from heavy CsCl gradient fractions exhibited greatly reduced richness and evenness relative to unlabeled DNA, indicating strong ^13^C-enrichment of *p*HB-degrading subpopulations (Fig. S5). Between 10 and 40% of all amplicon sequences in ^13^C-DNA pools corresponded to phylotypes classified as *Paraburkholderia* (*n* = 8) and *Caballeronia* (*n* = 4), of which six predominated in all ecoplots (Fig. 5A, B). Most of these phylotypes were also enriched by *p*HB addition in the microcosm priming experiment, including four of the six major phylotypes (Table S7). Two *Paraburkholderia* phylotypes, matching *P. madseniana* (RP11^ASV^) and *P. xenovorans* LB400, were the dominant *p*HB-responders in both soil microcosm priming experiments and DNA-SIP experiments performed in field soils (Figs. 2 and  5).

Less abundant ^13^C-*p*HB-labeled phylotypes detected in the DNA-SIP experiment included *Burkholderia*, *Collimonas*, *Pseudomonas* (Gammaproteobacteria), *Phenylobacterium* (Alphaproteobacteria), *Streptomyces* and Conexibacteraceae (Actinobacteria), and *Solicoccozyma* (a basidiomycotal yeast; Fig. S6). These phylotypes were observed sporadically among forest and soil types (Fig. 5B; Table S7), unlike phylotypes of *Paraburkholderia* and *Caballeronia* which predominated following *p*HB addition in all ecoplots.

### Isolation of pHB-degrading bacteria

Nine bacteria were isolated from RP_LnC_ soil using minimal media with *p*HB as the sole carbon source. All of these isolates produced protocatechuate (the reaction product of PobA) during growth on *p*HB (data not shown). Isolates were classified as *Paraburkholderia* (*n* = 5), *Pseudomonas* (*n* = 3) and *Cupriavidus*. Four of the *Paraburkholderia* isolates had identical full-length 16S rRNA genes that matched one of the dominant *p*HB-degrading phylotypes (RP11^ASV^) detected in DNA-SIP and microcosm experiments. One of these strains, isolate RP11^T^, was chosen for genome sequencing. The RP11^T^ genome was large (10.1 Mb), contained two chromosomes and encoded 6 copies of the *rrn* operon.^54^ The genome encoded the complete pathway to mineralize *p*HB and included two paralogs of *pobA* which shared 48% amino acid sequence identity. RP11^T^ encoded an array of oxidative enzymes, including a DyP-type peroxidase, laccases, aryl-alcohol oxidases and an array of ring-cleaving and ring-hydroxylating dioxygenases (Table S8). Several of these enzymes contained signal peptide sequences, indicating potential for extracellular activity, including the DyP-type peroxidase, three laccases, and an aryl-alcohol oxidase.^79^

### Phylogenetic diversity and environmental distribution of pobA-encoding bacteria

**Fig. 6:**
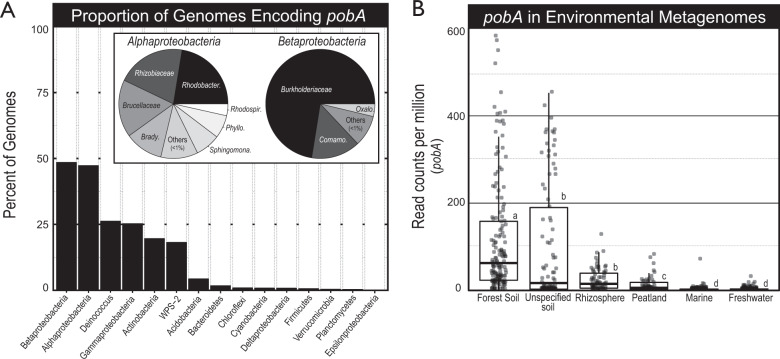
The phylogenomic distribution and environmental associations of *pobA* in (**A**) bacterial genomes (*n* = 11,643) and (**B**) environmental metagenomes (*n* = 14,139) from the IMG/ER database. In (**A**), phyla were ranked by the proportion of genomes encoding *pobA*. Only phyla represented by >10 genomes were surveyed, and only those with at least one genome encoding *pobA* are shown. The taxonomic classification of *pobA*-encoding genomes from *Alpha*- and *Betaproteobacteria* are indicated by the inset pie charts in (**A**). Each taxonomic group was normalized to the total number of genomes present in the database. Homologs of *pobA* were far more common in soils in general, and in forest soils in particular, relative to other sample types as shown in (**B**). Letters denote significant differences (*p* < 0.05) based on pairwise Wilcoxon Rank Sum tests.

In the IMG-ER genome database, homologs of *pobA* were most commonly encoded in genomes of *Betaproteobacteria* and *Alphaproteobacteria*, occurring in more than 45% of genomes classified to these groups (Fig. 6A, and Table S9). Genomes encoding paralogs (two copies of *pobA*) were more common in *Paraburkholderia* (25% of genomes) than any other genus of *pobA*-encoding *Burkholderiaceae* (Fisher’s exact, O.R. = 3.1, *p* < 0.001). In addition, *pobA* paralogs were more common in soil isolates compared to other environmental sources (O.R. = 2.7, *p* < 0.001; Fig. S7A). Furthermore, *pobA* homologs were more abundant in forest soil metagenomes than in other environmental sources, and this difference was significant (Fig. 6B). Homologs of *pobA* comprised seven major phylogenetic clades, and the two largest of these clades were represented by *pobA1* and *pobA2* of RP11^T^ (clades 6 and 5, respectively, Fig. S7B). Additional information on the phylogenetic diversity and inferred activity of *pobA* homologs can be found in the Supplementary Results.

## Discussion

Our results demonstrate that the degradation of *p*-hydroxybenzoic acid (*p*HB) can prime the decomposition of SOC in a variety of forest and soil types. This phenomenon was driven by the activity of *Paraburkholderia* and, to a lesser extent, *Caballeronia*, from the family *Burkholderiaceae*. The predominant *Paraburkholderia* phylotypes were related to *P. madseniana* RP11^T^ and *P. xenovorans* LB400^T^, species noted for having more genes and pathways for the degradation of phenolics and aromatics than close relatives,^54^ and among the highest of any bacterial species.^80^ Based on these observations, we conclude that the mechanisms underlying phenolic- and glucose-induced priming differ, since the former is driven by a closely related group of bacteria with marked functional specialization and the latter by the general activity of diverse populations.^47^ Repeated dosing of glucose is commonly required to cause priming,^47^ with a single bolus often resulting in negligible or negative priming, consistent with our observations.^15,81–83^ In contrast, a single dose of *p*HB (Fig. 1), benzoic acid^15^ or vanillin^14,16^ resulted in positive priming and the enrichment of *Paraburkholderia* in forest, agricultural and tundra soils, respectively. The identification of the taxa and functional genes responsible for phenolic-induced priming is an important step forward in understanding the types of microbial activity responsible for enhancing losses of SOC. Indeed, several lines of evidence in our study suggest phenolic acid-degrading bacteria play a general role SOC cycling, such as the correspondence between SOC content among tree types in our common garden and rates of phenolic acid-degrading activity.

### Characteristics of pHB-induced soil priming

The catabolism of *p*HB and glucose differed in ways that revealed differences in the nature of priming. The addition of *p*HB to soil yielded significant positive priming with nearly twice the amount of substrate mineralized compared to glucose, which yielded slight, though insignificant, negative priming (Fig. 1). This difference in substrate use efficiency has been documented in forest soils, where added *p*HB was mineralized at twice the rate of glucose, and produced only half the microbial biomass.^84^ The same effect occurred in grassland soil amended with vanillic acid, which yielded higher mineralization rates and lower microbial biomass than glucose or cellobiose, and also a greater positive priming effect by energy content.^14^ We hypothesize that *p*HB-induced priming is caused, in part, by a stoichiometric imbalance whereby C catabolism greatly exceeds the needs of anabolism, evident in the high rates of *p*HB mineralization. Stoichiometric limitation-induced degradation of soil organic matter is a well-known mechanism in priming.^47,49,81,85,86^ This could also explain why the turnover of RP11^T^ cells (a relatively rich nutrient source) was associated with strong suppression of SOC mineralization (Fig. S3). We propose that variation in the priming effect can be governed by substrate-specific metabolic use efficiency, due to differences in stoichiometric imbalances promoted during metabolism. These dynamics are governed by life-history traits of decomposer populations or competitive and facilitative interactions with other soil microbes which are not yet fully understood.

Growth rate is a common determinant of substrate use efficiency and, in our case, may contribute to the high mineralization rates of *p*HB. *p*HB-degrading populations were dominated by fast-growing bacteria which tend to be less efficient in converting substrate C into biomass.^87^ Members of the family *Burkholderiaceae* are major zymogenous populations in soil^35^ and the rapid in situ growth of RP11^ASV^ was evident in its increase from ~0.7 to 15% of total sequences in 24 h, during the period of maximal *p*HB respiration. Consistent with these observations, isolate RP11^T^ exhibited rapid growth (*µ* = 0.22 h^−1^) in pure culture with *p*HB as the sole carbon source,^54^ comparable to the specific growth rate of *E. coli* on acetate (*µ* = 0.18 h^−1^).^88^ The RP11^T^ genome also encoded six copies of the *rrn* operon, a characteristic of high growth rate.^89^ These observations suggest that rapid growth is a microbial trait relevant to priming, highlighting the importance of pulsed sources of phenolic C in SOC cycling.

**Fig. 7:**
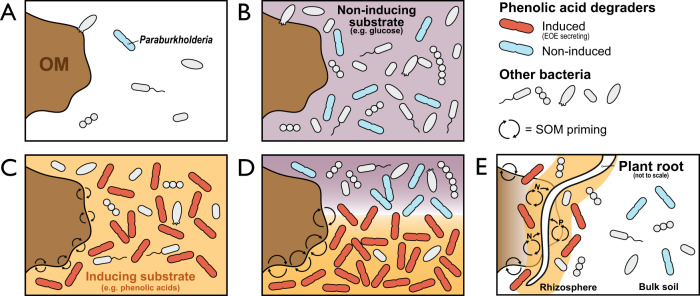
A conceptual framework illustrating the dependency of SOM priming on the abundance of phenolic acid-degrading bacteria and the induction of genes encoding extracellular oxidative enzymes (EOE) that degrade aromatic and polyaromatic compounds. In (**A**), limiting SOM priming occurs when phenolic acid-degrading bacteria are rare and aromatic metabolism is not induced. In (**B**), the addition of non-inducing substrates, such as glucose, stimulate growth of aromatic acid-degrading bacteria, but priming does not occur if the concentration of aromatic acids is insufficient to induce EOE. In (**C**), the addition of inducing substrates, such as phenolic or other aromatic acids, stimulate SOM priming by inducing EOE and by promoting the growth of specialized degrader populations. In (**D**), if the concentration of endogenous aromatic acids is sufficient to induce EOE then the co-addition of non-inducing substrates can enhance SOM priming by promoting the growth of phenolic acid-degrading bacteria. Hence, while glucose can stimulate the growth of *Paraburkholderia*, priming only occurs when aromatic metabolism is induced. In (**E**), plant roots gain access to inaccessible forms of N and P by stimulating the growth and induce priming activity of phenolic acid-degrading bacteria by exuding phenolic/aromatic acids.

Priming was also contingent on the metabolic state of degrader populations. Identical *Paraburkholderia* phylotypes responded to both glucose and *p*HB additions in RP_LnC_ and BL_LnC_ (Fig. 2), yet positive priming was induced only by *p*HB. This phenomenon was apparent in a related study on soil priming, where the same *Paraburkholderia* phylotypes responded to benzoic acid and glucose amendment, yet only benzoic acid elicited positive priming.^15^ Notably, the co-addition of glucose and benzoic acid enhanced priming and coincided with an even greater increase in *Paraburkholderia* (identical to our two major phylotypes). These observations are supported by the regulation of oxidative catabolic pathways in *Paraburkholderia* by aromatic and phenolic compounds, which are insensitive to glucose concentration.^90,91^ We conclude that the regulation of metabolic state is critical for priming by phenolic-degrading populations, which may be enhanced, but not triggered, by glucose or other non-inducing substrates (Fig. 7).

The activity of oxidative enzymes has long been considered a potential mechanism in priming, resulting from the co-metabolism of SOC.^11,81^ *Paraburkholderia* are renowned for their oxidative degradation of aromatics, phenolics, and polyaromatic hydrocarbons.^92,93^ The two dominant *p*HB-degrading phylotypes in our study matched to *P. madseniana* and *P. xenovorans*, species which encode an extensive array of aromatic degrading pathways.^54,94^ *P. madseniana* RP11^T^ encoded among the greatest number of aromatic degrading genes compared to close relatives, including several putatively secreted oxidases^79^ and aryl-alcohol oxidases important for bacterial lignin degradation.^42^ *P. madseniana* RP11^T^ also grows on phthalic acid, a major by-product of lignin degradation.^95–97^ Indeed, our retrospective analysis found that the two major *p*HB-degrading phylotypes of *Paraburkholderia* predominated in studies of lignin degradation^42^, and white rot decay^43^, and were the principal phylotypes in all phenolic/aromatic-based studies of soil priming^14–16^ (see Supplementary Results). All evidence indicates that *Paraburkholderia*, and their close relatives, cause priming by monopolizing phenolic acid degradation and co-metabolizing SOC.

### Impacts of phenolic acid-degrading populations on soil C-cycling

*Paraburkholderia* and *Caballeronia* routinely occur where lignin- and phenolic-rich plant matter is decomposed,^43,44,98–100^ and in association with plant roots,^101^ where aromatic- and phenolic-rich exudates can recruit *Paraburkholderia* during development.^102,103^ The capacity for phenolic acids to prime decomposition, and abundance of plant-derived phenolic sources to soil, raises questions about the potential impact of this phenomenon on soil C-cycling. Phenol oxidase activity and SOC content are inversely correlated at ecosystem scales, but this relationship is weak and highly interrelated with other soil properties.^104^ In our field experiment, *p*HB-degrading activity and total SOC content were inversely proportional. *p*HB-degrading activity was greatest where the relative abundance of *Paraburkholderia* and *pobA* expression were highest, and where SOC was lowest (SM_LnC_ and RP_LnC_). These observations raise the prospect that phenolic-induced priming may influence forest SOC cycling at scale. However, we interpret these results with caution, given that a single time point cannot capture the full extent of processes contributing to differences in SOC in our 70-year-old common garden. SOC content also followed trends in soil pH, iron, nickel and arsenic content which could independently affect SOC accrual. Still, the potential for plant-derived phenolics to influence SOC formation via priming is notable, given the biology and ecology of the phenolic acid-degrading populations.

*Paraburkholderia* and *Caballeronia* can exist as endophytes in a broad range of plants^105–110^ and are capable of nodulating legumes, including black locust.^111^ All three of the closest related genomes to RP11^T^ were isolated from plant roots: *P. sycomorum* ST111 (94.3% ANI; from *Acer* roots)^112^, *P. aspalathi* LMG 27731^T^ (94.2%; *Aspalathus* root nodule)^113^ and *P. sp*. OK233 (94%; *Populus* roots).^114^ The capacity to form associations with roots suggests that rhizosphere stimulation may underlie differences in *p*HB-degrading activity among tree types and why differences in *p*HB-degrading activity were observed in situ and not in microcosms (i.e., absent of root influence). The highest in situ *p*HB-degrading activity was recorded in SM plantations where root density was by far greatest, according to field observations (Fig. S1) and prior research.^32^ In contrast, soils in BL plantations exhibited the lowest *p*HB-degrading activity and were comprised of different *Burkholderiaceae* populations (Fig. 2). Black locust was the only leguminous tree species in our common garden capable of obtaining nitrogen through internal root symbioses, indicating differences in phenolic acid-degrading activity and priming may result from different relationships between plant and microbe. Phenolic root exudates are known to facilitate plant-microbe interactions that can be essential for plant nutrition.^45,115^ For instance, *p*HB concentrations doubled in exudates of poplar trees grown in nitrogen and phosphorus limiting conditions.^31^ We hypothesize that certain tree species may modulate the phenolic acid content of exudates to stimulate priming and gain access to nutrients stored in soil organic matter (Fig. 7E).

### Tracking priming activity with pobA

The expression of *pobA* was consistent with *p*HB-degrading activity and reflected the transient nature of *p*HB-induced priming. Tracking priming activity based on the expression of functional genes can discern between mere demographic changes in populations associated with priming and the metabolic states associated with real priming activity. In our study, the relative abundance of *Paraburkholderia* increased when either glucose or *p*HB were added but priming occurred exclusively in the latter instance. Changes in the relative abundance of *pobA* in soil metagenomes was also coincident with vanillin-induced priming, and an increase in the *P. xenovorans* phylotype.^16^ Together, these findings illustrate the potential utility for tracking priming dynamics using *pobA*—a key enzyme in the peripheral degradation of phenolics.^51^

## Conclusions

We conclude that specialized bacteria from the family *Burkholderiaceae* act as principal agents of phenolic acid-induced soil priming in forest soils, and likely more broadly. These populations consisted primarily of fast-growing *Paraburkholderia* characterized by their capacity to produce strongly oxidative catabolic enzymes, and to associate with plant roots. Our study builds on our prior work showing the priming activity of *Paraburkholderia,*^15^ and characterizing the predominant phenolic acid degrading isolate as a new species,^54^ by providing the essential microbiological evidence for understanding their ecology and function in priming. The transient nature of *p*HB-induced priming underscores the importance of pulsed resource availability, or “hot moments”, in soil C-cycling,^116^ and the utility of dialing in on functional gene expression. Moving forward, it is imperative to characterize the role of phenolic acid-degrading activity and priming in natural settings where seasonal and root-mediated dynamics are considered. Even slight environmental changes can lead to changes in priming, as evidenced by the effect of warming on phenolic acid-induced degradation of recalcitrant SOC by *Paraburkholderia* in tundra soil.^16^ Future studies of phenolic acid-induced priming might help shed light on the nature of SOC persistence, which has previously been linked to low phenolic acid content.^19,20^

## Supplementary information

Supplementary Methods

Supplementary Results

Supplementary Figures

Supplementary Tables

Supplementary Data
