# Supplementary material for: Phenolic acid-degrading Paraburkholderia prime decomposition in forest soil
Source: ISME Commun. 2021 Mar 25;1:4. doi: 10.1038/s43705-021-00009-z (PMC9723775; doi:10.1038/s43705-021-00009-z)
Supplement: Supplementary file 2 — Supplementary Results [file 43705_2021_9_MOESM2_ESM.pdf]

Supplementary Results

From Wilhelm and DeRito *et al.*, 2021 – Phenolic acid-degrading populations of *Paraburkholderia* prime decomposition in forest soils.

PobA inhibition of RP11^T growth on pHB

The effective dose of methyl 4-hydroxy-3-iodobenzoate (m-*p*HB), an inhibitor of PobA, was determined by measuring the growth of *P. madseniana* RP11^T on *p*HB in the presence of a dilution gradient of m-*p*HB. The MSM+*p*HB medium was prepared with varying concentrations of m-*p*HB: 0.486 mM (saturated solution at RT), 0.486 mM x 10^{-1}, 0.486 mM x 10^{-2}, 0.486 mM x 10^{-3} and 0.486 mM x 10^{-4}. Growth was measured by OD. All concentrations of m-*p*HB initially resulted in decreased growth, but only the saturated concentration inhibited growth below detection, relative to uninoculated controls. In all cases, growth was eventually observed with the saturated concentration suppressing growth completely for 4 days.

Re-analyzing published 16S rRNA gene amplicon libraries

16S rRNA gene amplicon data from several studies reporting the involvement of '*Burkholderia'* in white-rot [1], lignin-degradation [2], soil priming [3–5] or plant roots [6–9] were re-analyzed and determined to consist primarily of *Paraburkholderia* with dominant populations matching to *P. madseniana* RP11^T and *P. xenovorans* LB400^T in all but [7]. The majority of this information was published in Supplementary Table 5 in Wilhelm *et al.* [10].

Structure-function relationships of pobA paralogs

The paralogs of *pobA* in RP11^T were distinct and homologous to structurally characterized enzymes from *Pseudomonas CBS3* [11] (67% identity to *pobA1*) and *P. fluorescens* [12] (76% to *pobA2*). Key residues involved in *p*HB-binding (Ser_{212}, Arg_{214} and Tyr_{222}) were conserved in both paralogs, but *pobA1* and *pobA2* differed in a residue involved in flavin-binding that can alter affinity for NADPH (Arg_{42}) versus NADH (Thr_{42}), respectively [13]. We propose several hypotheses about the role of the putative NADPH-consuming PobA, given that NADPH is primarily used in anabolism. One hypothesis is that this NADPH dependent PobA paralog modifies *p*-hydroxybenzoate as part of an uncharacterized biosynthetic pathway. Alternatively, the *pobA* paralog might serve in a *luxR* dependent sense and response system as described for *p*-coumaric acid, which is hypothesized to enable bacterial-plant signalling [14]. RP11^T encodes *luxI* and *luxR* homologs and endophytic *Paraburkholderia* from poplar roots (isolate BT03) do exhibit quorum sensing activity [15]. It is also possible that the NADPH dependent *pobA* paralog might function in detoxification or O_2 scavenging in the cytoplasm. For example, *Paraburkholderia* are obligate aerobes that are often capable of nitrogen fixation [16], and O_2 scavenging enzymes could serve to protect nitrogenase from inactivation by O_2. The use of dioxygenases in O_2 scavenging has been shown to occur in phenolic acid-rich termite guts [17].

Evolutionary reconstruction of pobA paralogs

The existence of *pobA* paralogs in RP11^T prompted an investigation into the identity, relatedness and environmental associations of *Burkholderiaceae* encoding paralogs. *Paraburkholderia* were likelier to encode paralogs than all other *pobA*-encoding *Burkholderiaceae* (Fisher's exact, O.R. = 3.1, $p < 0.001$). Twenty-five percent of *Paraburkholderia* encoded paralogs compared to the next highest frequency in *Burkholderia* (9%). Paralogs of *pobA* were most common in soil isolates compared to other environmental sources (Fisher's exact, O.R. = 2.7, p <

0.001; Figure 7A). The phylogenetic depth at which paralogs occurred within related groups of *Burkholderiacaea* was relatively shallow (τ_D = 0.005 out of a total depth of 2.5 units) compared to other aromatic degradation genes, such as gentistate 1,2-dioxygenase (τ_D = 0.009), benzoate 1,2-dioxygenase (τ_D = 0.01) and protocatechuate 3,4-dioxygenase (τ_D = 1.0; Table S2). Genomes encoding paralogs were the most phylogenetically clustered of all aromatic degrading genes tested, exhibiting a Brownian mode of trait inheritance (Purvis and Fritz's D = 0.01) in contrast to the more random dispersion pattern of the protocatechuate dioxygenase gene (D = 0.249; Table S2). Notably, 32% of *pobA*-encoding actinobacterial genomes encoded multiple copies: commonly two (21%) or three (8%), but also up to five in *Streptomyces* and *Amycolatopsis* genomes (~1%). No *Burkholderiaceae* genome encoded more than two copies.

Here is useful context for interpreting values of D: a value of $D < 0$ suggests a highly clustered trait, $D \sim 0$ indicates a Brownian motion mode of evolution, $D = 1$ suggests a random mode of evolution and $D > 1$ suggests phylogenetic overdispersion. Martiny et al. [18] write "Complex traits encompassing many genes like oxygenic photosynthesis and methanogenesis were extremely clumped ($D << 0$). Other traits including nitrogen and CO_2 fixation, anoxygenic photosynthesis and sulfate reduction displayed a clumped distribution consistent with a Brownian motion model of evolution ($D \sim 0$). Finally, traits like carbon substrate utilization were dispersed in a mode between a Brownian motion and a random model ($0 < D > 1$), suggesting that the ability to grow on different carbon compounds is quite dispersed."

Targeted assembly of pobA from published metagenomes

A targeted assembly of *pobA* was performed on metagenomes constructed from ^{13}C-enriched or ^{12}C-control DNA derived from forest soil incubations with either ^{13}C-lignin or natural abundance ^{13}C lignin [2] (methods in Supplementary methods). The assembly yielded 1,204 *pobA* homologs occurring exclusively in metagenomes derived from ^{13}C-enriched DNA pools (Table S3). The majority of *pobA* recovered belonged to clade 5 (82%), followed by clade 4 (9.4%) and clade 6 (6.7%). The *pobA* sequences were most related to *Rhizobiales* (44%), *Burkholderiales* (41%), *Sphingomonadales* (10%) and *Pseudomonadales* (4%) according to Lowest Common Ancestry. Six of the most abundant amplicon phylotypes from [2] were identical to the 16S rRNA gene of *P. madseniana* $RP11^T$. However, none of the recovered *pobA* were identical to paralogs in $RP11^T$ (top % identity according to BLASTp = 71% for *pobA.1* and 75% for *pobA.2*).

Mechanism of negative priming

The addition of stimulated $RP11^T$ cells to soils was designed to test whether priming would be induced by an increased presence of *p*HB-degrading bacterium. Contrary to expectations, the addition of $RP11^T$ biomass produced a net decrease in respiration of existing SOC of between 5 - 14% relative to controls (i.e. negative priming), which showed no signs of abating by the end on day 26. Negative priming was associated with steep increases in the relative abundance of *Streptomyces* and *Streptacidiphilus* phylotypes (Figure S3C). These populations were below detection in field soil (min_{reads} = 7,318) and increased to upwards of 35% of the total amplicon pool in $RP11^T$-amended soil. The trend in their relative abundance was more pronounced in cDNA than DNA pools, indicating their increased representation in active populations at day 7 (Figure S3D).

We attribute the negative priming to the preferential use of $RP11^T$ biomass by decomposers in place of soil organic matter. Soils were amended with $RP11^T$ cells at a relatively low abundance to mirror a rare taxon in soil (~ 0.001% of total bacteria). Our experiment was the first attempt to

use cell biomass in a priming study and the surprising strength of negative priming was unanticipated. We believe the negative priming overshadowed any positive priming which may have occurred due to the addition of stimulated RP11^T cells. *Streptomyces* may produce antibiotics or ammonia [19] that lowers net decomposer activity or may increase the net efficiency of organic matter degradation. Their growth in glucose-amended soils was consistent with the expected greater proportion of glucose being converted to biomass. The effect of biomass in stimulating populations that actively diminish the mineralization of SOC suggests the physical 'entombing' of biomass [20] is not the only mechanism by which biomass influences SOC cycling.

References

1. Folman LB, Klein Gunnewiek PJA, Boddy L, De Boer W. Impact of white-rot fungi on numbers and community composition of bacteria colonizing beech wood from forest soil. *FEMS Microbiol Ecol* 2008; **63**: 181–191.
2. Wilhelm RC, Singh R, Eltis LD, Mohn WW. Bacterial contributions to delignification and lignocellulose degradation in forest soils with metagenomic and quantitative stable isotope probing. *ISME J* 2019; **13**: 413–429.
3. Lonardo DP Di, Boer W De, Gunnewiek PJAK, Hannula SE, Wal A Van Der, Di Lonardo DP, et al. Priming of soil organic matter: chemical structure of added compounds is more important than the energy content. *Soil Biol Biochem* 2017; **108**: 41–54.
4. Zwetsloot MJ, Muñoz Ucros J, Wickings K, Wilhelm RC, Sparks JP, Buckley DH, et al. Prevalent root-derived phenolics drive shifts in microbial community composition and prime decomposition in forest soil. *Soil Biol Biochem* 2020; **145**.
5. Tao X, Feng J, Yang Y, Wang G, Tian R, Fan F, et al. Winter warming in Alaska accelerates lignin decomposition contributed by Proteobacteria. 2020; 1–12.
6. Thijs S, Van Dillewijn P, Sillen W, Truyens S, Holtappels M, D´Haen J, et al. Exploring the rhizospheric and endophytic bacterial communities of *Acer pseudoplatanus* growing on a TNT-contaminated soil: towards the development of a rhizocompetent TNT-detoxifying plant growth promoting consortium. *Plant Soil* 2014; **385**: 15–36.
7. Zhalnina K, Louie KB, Hao Z, Mansoori N, da Rocha UN, Shi S, et al. Dynamic root exudate chemistry and microbial substrate preferences drive patterns in rhizosphere microbial community assembly. *Nat Microbiol* 2018; **3**: 1–11.
8. Haeckl FPJ, Baldim JL, Iskakova D, Kurita KL, Soares MG, Linington RG. A selective genome-guided method for environmental *Burkholderia* isolation. *J Ind Microbiol Biotechnol* 2019; **46**: 345–362.
9. Shiraishi A, Matsushita N, Hougetsu T. Nodulation in black locust by the Gammaproteobacteria Pseudomonas sp. and the Betaproteobacteria Burkholderia sp. *Syst Appl Microbiol* 2010; **33**: 269–274.
10. Wilhelm RC, Murphy SJL, Feriancek NM, Karasz DC, Derito CM, Newman JD, et al. *Paraburkholderia madseniana* sp. nov., a phenolic acid-degrading bacterium isolated from acidic forest soil. *Int J Syst Evol Microbiol* 2020; 10.1099/ijsem.0.004029.
11. Seibold B, Matthes M, Eppink MHM, Lingens F, Berkel W, Muller R. Purification, characterization, gene cloning, sequence analysis and assignment of structural features determining the coenzyme specificity. *Eur J Biochem* 1996; **239**: 469–478.
12. van Berkel WJH, Westphal AH, Eschrich K, Eppink M, de Kok A. Substitution of Arg214 at the substrate-binding site of *p*-hydroxybenzoate hydroxylase from *Pseudomonas fluorescens*. *Eur J Biochem* 1992; **210**: 411–419.

13. Eppink MHM, Schreuder HA, Van Berkel WJH. Lys42 and Ser42 variants of *p*-hydroxybenzoate hydroxylase from *Pseudomonas fluorescens* reveal that Arg42 is essential for NADPH binding. *Eur J Biochem* 1998; **253**: 194–201.
14. Schaefer AL, Greenberg EP, Oliver CM, Oda Y, Huang JJ, Bittan-Banin G, et al. A new class of homoserine lactone quorum-sensing signals. *Nature* 2008; **454**: 595–599.
15. Schaefer AL, Lappala CR, Morlen RP, Pelletier DA, Lu TYS, Lankford PK, et al. LuxR- and luxI-type quorum-sensing circuits are prevalent in members of the populus deltoides microbiome. *Appl Environ Microbiol* 2013; **79**: 5745–5752.
16. Chan Y. Utilization of simple phenolics for dinitrogen fixation by soil diazotrophic bacteria. *Plant Soil* 1986; **90**: 141–150.
17. Lucey KS, Leadbetter JR. Catechol 2,3-dioxygenase and other meta-cleavage catabolic pathway genes in the 'anaerobic' termite gut spirochete *Treponema primitia*. *Mol Ecol* 2014; **23**: 1531–1543.
18. Martiny AC, Treseder K, Pusch G. Phylogenetic conservatism of functional traits in microorganisms. *ISME J* 2013; **7**: 830–838.
19. Avalos M, Garbeva P, Raaijmakers JM, van Wezel GP. Production of ammonia as a low-cost and long-distance antibiotic strategy by *Streptomyces* species. *ISME J* 2019; 569–583.
20. Liang C, Schimel JP, Jastrow JD. The importance of anabolism in microbial control over soil carbon storage. *Nat Microbiol* 2017; **2**: 1–6.
