# Supplementary figures and images for: Phenolic acid-degrading Paraburkholderia prime decomposition in forest soil
Source: ISME Commun. 2021 Mar 25;1:4. doi: 10.1038/s43705-021-00009-z (PMC9723775; doi:10.1038/s43705-021-00009-z)

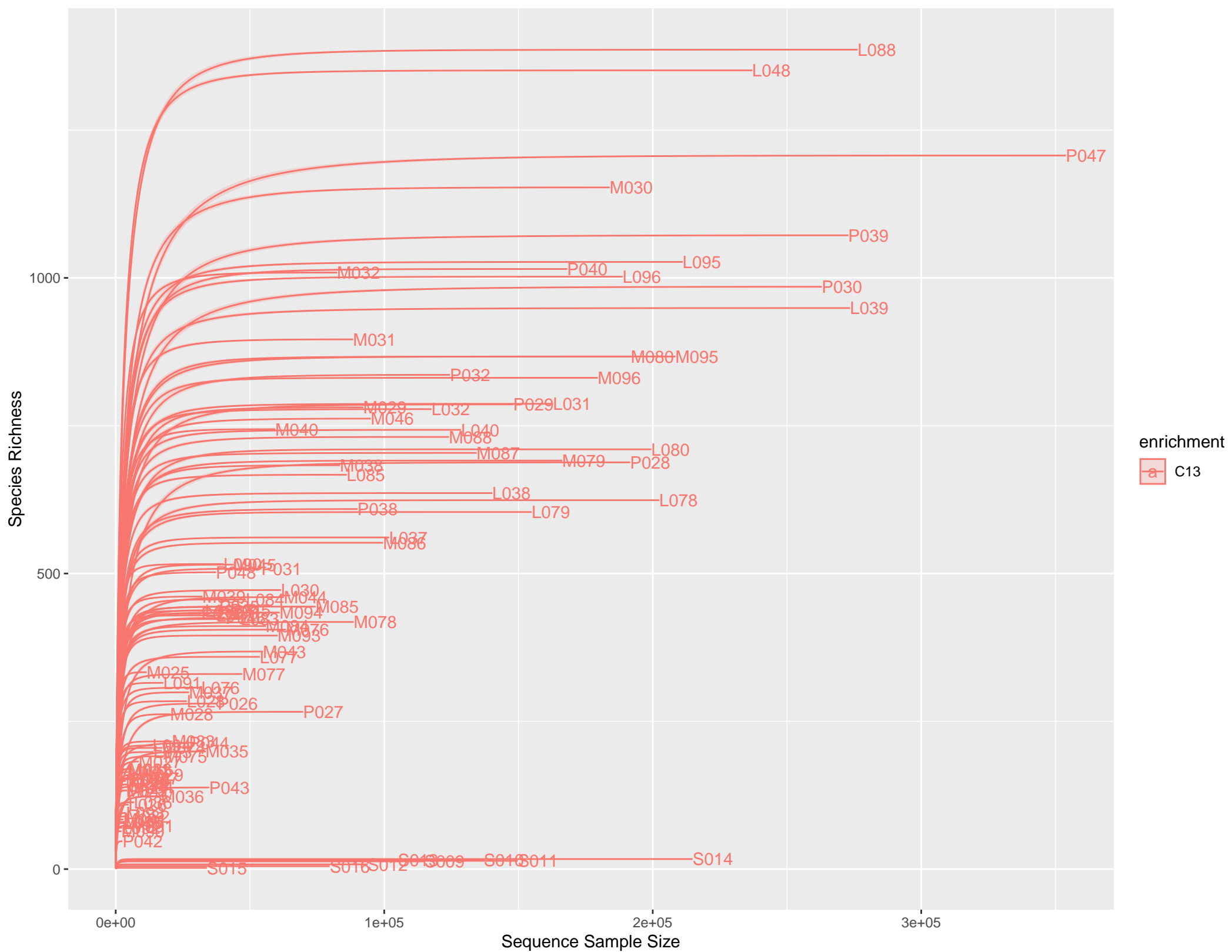

Supplement: Supplementary file 5 — Supplementary Data [file 43705_2021_9_MOESM5_ESM.zip › Supplementary Data/3. Rarefaction curves - SIP amplicon libraries.pdf]
